# Predictors of recurrent ischemic stroke: anatomical characteristics of patent foramen ovale

**DOI:** 10.3389/fcvm.2025.1658804

**Published:** 2025-11-14

**Authors:** Xiaoyan Chen, Jianxiu Fang, Qingmei Yang, Liping Liu

**Affiliations:** 1 Department of Ultrasound, Shanxi Bethune Hospital, Shanxi Academy of Medical Sciences, Third Hospital of Shanxi Medical University, Tongji Shanxi Hospital, Taiyuan, China; 2 Department of Interventional Ultrasound, First Hospital of Shanxi Medical University, Taiyuan, China

**Keywords:** cryptogenic stroke, patent foramen ovale, recurrent, transesophageal echocardiography, transthoracic echocardiography

## Abstract

**Background:**

Percutaneous closure of patent foramen ovale (PFO) is effective in preventing PFO-related stroke recurrence. Considerable further research aimed at acquiring relevant data is needed to enable reliable identification of the subgroup of patients with PFO who would benefit from such closure.

**Objectives:**

This study aimed to select optimal candidates for PFO closure; we used echocardiography to assess various anatomical features of PFO, and then determined the value of those features for predicting recurrent PFO-related stroke.

**Methods:**

We used echocardiography to analyze selected anatomical characteristics of PFO, including atrial septal aneurysms (ASA), right-to-left shunts, PFO width, and tunnel length, in patients with PFO-related stroke. The participants underwent PFO closure, mostly plus medication, or medication-only and were followed up for at least 2 years. We compared the rates of recurrent stroke within 2 years between the two groups, and the anatomical characteristics of PFO between patients with and without recurrent stroke using mixed effects Cox models.

**Results:**

The study comprised 207 patients (120 males, 54.17%). Percutaneous PFO closure was successfully performed in 120 patients (PFO closure group), while 87 received medication-only therapy (medication-only group). After 2 years of follow-up, the risk of recurrent PFO-related ischemic stroke was was significantly lower in the PFO closure group (0.83%) than in the medication-only group (9.2%). The combination of anatomical features of ASA and large right-to-left shunts was associated with recurrent PFO-related stroke. Presence of an ASA alone was independently associated with recurrent PFO-related stroke (B = 2.43, hazard ratio = 11.37; 95% CI: 1.53–85.14; *P* = 0.012).

**Conclusions:**

The anatomical features of ASA and large right-to-left shunts are associated with recurrent PFO-related stroke in patients with PFO. These patients may derive the most benefit from PFO closure because of the associated greater reduction in absolute risk.

## Introduction

Approximately a quarter of adults have a patent foramen ovale (PFO), PFOs being detected in 15%–25% of patients undergoing echocardiography and 15%–35% of autopsies (1–5). PFOs are associated with many neurological disorders, including cryptogenic stroke (CS), migraine, obstructive sleep apnea, and decompression disorders (6–12). Ischemic stroke is the most common type of PFO-related neurological disorder. Epidemiological studies have shown a prevalence of PFO of 40%–50% in patients with CS (11–13). Compared with healthy people, individuals with PFO have a higher incidence of stroke (14). The paradoxical embolism that can occur through a PFO is hypothesized to be a leading cause of CS, especially in younger patients, who are otherwise at low risk of this disorder. Therefore, in patients, especially young patients, with CS, PFO may be a risk factor for recurrent stroke. Whether or not a PFO should be closed to prevent recurrent strokes in patients diagnosed with CS has long been controversial. The concept of “PFO-related stroke” was proposed in the Society for Cardiovascular Angiography and Interventions guidelines in 2022. These guidelines recommend PFO closure to prevent stroke recurrence in patients aged 18–60 years with PFO-related stroke (15). Percutaneous PFO closure has been shown to generally be effective in preventing PFO-related stroke recurrence (16–18). However, PFO closure is relatively ineffective in patients with long tunnel PFO (19). In addition, atrial fibrillation occurs more frequently in patients who have undergone PFO closure than in those receiving medical therapy alone (20). Therefore, considerable further research aimed at acquiring relevant data is needed to enable reliable identification of the subgroup of patients with PFO who would benefit from PFO closure (21).

Certain anatomic characteristics of PFO have been shown to predict the risk of recurrent PFO-related stroke and provide evidence to justify PFO closure (22–25). Transesophageal echocardiography (TEE) combined with contrast echocardiography is the recognized gold standard for noninvasive diagnosis of PFO. These procedures quantitatively assess the morphologic features of PFOs, semi-quantitatively assess right-to-left shunting, and can measure atrial septal aneurysms (ASA) (26, 27).

In this study, echocardiography was used to assess the morphologic features of PFO in patients with CS and included in follow-up of these patients after PFO closure plus drug therapy or drug therapy only. We also examined the correlation between recurrent PFO-related stroke and PFO morphology with the aim of determining the value of certain anatomical features of PFO in predicting recurrent PFO-related stroke in these patients, thus assisting selection of optimal candidates for PFO closure.

## Methods

### Subjects

**Figure 1:**
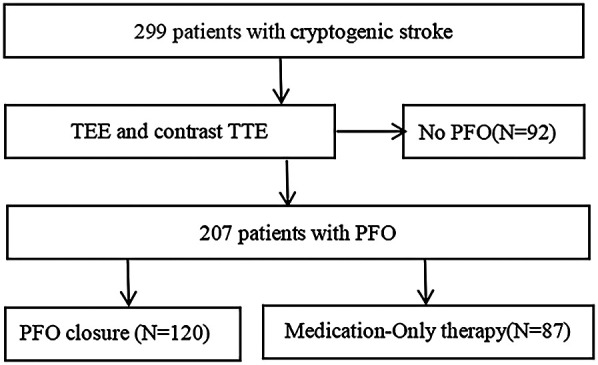
Flow diagram of the study. PFO, patent foramen ovale; TEE, transesophageal echocardiography; TTE, transthoracic echocardiography.

The study comprised 299 candidates who were less than 70 years old and had a history of a CS between January 2018 and December 2022. Among these patients, 207 (mean age, 46.42 ± 13.32 years; range, 14–69 years; 114 male and 93 female) had a PFO. Of these 207 patients, 120 underwent percutaneous PFO closure plus antiplatelet therapy (PFO closure group). All PFO closures were successful. The other 87 patients received only antiplatelet therapy (medication-only group), either because they refused to undergo PFO closure or because of comorbidities that contraindicated PFO closure (Figure 1).

PFO-related CS is defined as a non-lacunar brain infarction that remains cryptogenic after complete vascular, cardiac and laboratory work-up but is statistically or mechanistically linked to a patent foramen ovale. We diagnosed PFO-related CS by performing a stringent standardized examination to rule out other identifiable causes of stroke, such as large artery atherosclerotic disease, an established cardioembolic source, small-vessel occlusive disease, a hypercoagulable disorder requiring anticoagulation, or arterial dissection (28). (1) We defined large artery atherosclerosis as ≥50% stenosis or occlusion of the corresponding artery; (2) lacunar stroke as a small (≤20 mm-diameter), deep infarct with no arterial or cardiac sources of embolism; (3) causes of cardioembolism included atrial fibrillation, recent (within 4 months) myocardial infarction, dilated cardiomyopathy, rheumatic mitral stenosis, mitral or aortic vegetations or prostheses, left atrial or left ventricular thrombus or tumor, akinetic left ventricular segment, spontaneous echocardiographic contrast of the left atrium, and complex atheroma of the aortic arch; and (4) other definite causes of stroke included non-atherosclerotic arteriopathies (e.g., dissection), coagulopathies, and hematologic or systemic disorders (e.g., antiphospholipid-antibody syndrome).

All participants gave their written informed consent for all investigations. The informed consent form for the minor patient is signed by their legal guardian. This study was approved by the Institutional Ethics Committee of Shanxi Bethune Hospital, the ethical board approval number is YXLL-2019-026. The study adhere to the Declaration of Helsinki.

### Equipment and diagnostic procedure

In this study, Color Doppler ultrasound diagnostic apparatus (model: Philips EPIQ7C), which has aX8-2t transesophageal probe of 1–15 MHz frequency, was utilized to make the diagnoses. All patients were instructed to fast for more than 12 h, and removable dentures were removed before the examination. Before the TEE procedure, all patients received 2% lidocaine mucilage for oropharyngeal anesthesia for 10 min. Using both two-dimensional and color Doppler ultrasonography, a probe was rotated from 0°–180° to clearly display the primary and secondary septa and detect any PFOs.

### Anatomical features of PFOs

**Figure 2:**
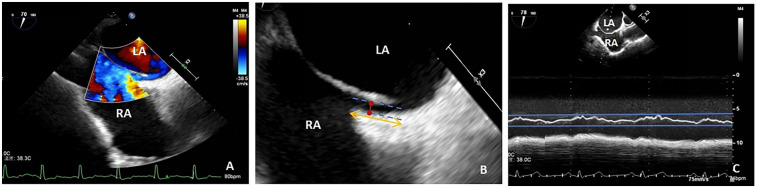
**(A)** TTE shows a diagonal right-to-left shunt between the left and right atria, diagnosed as patent foramen ovale (PFO); **(B)** PFO width, maximum distance between the primary and secondary septa (red arrow); PFO tunnel length, length of overlap between the septum primum and the secundum (yellow arrow); **(C)** atrial septal aneurysm, M-mode measurement of septum primum excursion ≥ 10 mm from the plane of the atrial septum into the right atrium; LA, left atrium; RA, right atrium; TEE, transesophageal echocardiography.

PFO width (maximum distance between primary and secondary septum) and tunnel length (length of overlap between primary and secondary septum) were measured using two-dimensional echocardiography and color Doppler multi-slice multi-angle observations (Figures 2A,B), whereas the amplitude of the atrial septum swing was measured using M-type echocardiography (Figure 2C). ASAs were defined as a septum primum excursion of ≥10 mm from the plane of the atrial septum into the right or left atrium, or swing amplitude ≥15 mm between left and right atria, with a base diameter of ≥15 mm (29). The data were averaged over three measurements taken by an echocardiographer who was blinded to the type of treatment the patient was to receive.

### Contrast transthoracic echocardiography (TTE)

**Figure 3:**
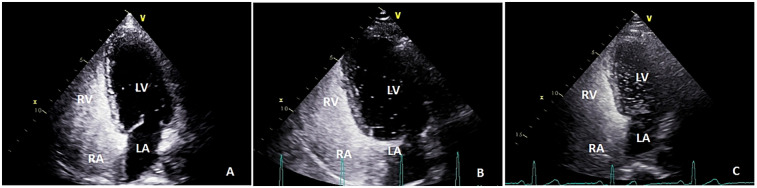
The number of microbubbles in left atrium (right-to-left shunt) during the five cardiac cycles after right atrial echocardiography was observed by transthoracic apical four-chamber view: **(A)** small (<10 microbubbles); **(B)** medium (10–30 microbubbles); and **(C)**, large (>30 microbubbles); LA, left atrium; RA, right atrium; LV, left ventricle; RV, right ventricle.

Color Doppler ultrasound diagnostic apparatus (model: Vivid™E9, which has an M5S probe frequency of 1.5–4.5 MHz) was utilized to examine right-to-left shunts. Saline microbubble contrast agent was prepared as follows: 8 mL of 0.9% saline was drawn into one 10 mL syringe and 1 mL of air into another 10 mL syringe, after which these syringes were connected to two of the ports of a medical three-way valve. The third port was connected to a scalp needle for cubital vein puncture. After successful puncture, about 1 mL of the examinee's venous blood was drawn into the syringe containing 8 mL of normal saline. After adjusting the three-way valve, the 1 mL of air and the 8 mL of normal saline and blood were thoroughly mixed by quickly pushing the syringes’ contents back and forth approximately 21 times and the resultant mixture injected into the patient's cubital vein. The patient was then placed in a left lateral decubitus position and injected with contrast medium at rest and performing the Valsalva maneuver, the interval between these two injections being more than 5 min. The number of microbubbles in the left atrium (right-to-left shunt) during the five cardiac cycles after right atrial echocardiography was counted via a transthoracic apical four-chamber view. The findings were classified as follows: negative, small (<10 microbubbles), medium (10–30 microbubbles) and large (>30 microbubbles) (30) (Figure 3). This procedure was carried out with TTE whenever possible because Valsalva maneuvers are easier to perform during TTE (31).

### Intra-observer and inter-observer reproducibility of echocardiographic measurements

PFO width was re-measured on the stored images of 10 randomly selected subjects by a second blinded observer; the intraclass correlation coefficient (ICC) was 0.92 (95% CI: 0.81–0.97), indicating excellent inter-observer reproducibility. For intra-observer assessment, the same observer re-analysed the same images in random order 1 week apart; ICC was 0.94 (95% CI: 0.86–0.98), demonstrating excellent intra-observer reliability.

### Percutaneous closure of a patent foramen ovale

After achieving local anesthesia with 1% lidocaine, a sheath tube was inserted into the femoral vein and heparin injected into it. An MPA2 angiography tube was then inserted into the right atrium along the sheath tube. Next, a Misgurnus anguillicaudatus guide wire was inserted through the sheath tube, passed through the patent foramen ovale into the left superior pulmonary vein, advanced via the catheter into the left atrium, and then further along the catheter into the left superior pulmonary vein, after which the MPA2 angiography tube was removed. After confirming by echocardiography that the guide wire had passed through the foramen ovale into the left atrium, it was inserted into the occluder sheath tube to the left atrium, and an appropriate occluder inserted along that tube. Pulling the sheath tube back opened the left atrial disc, closing the left atrial septum. It was then pulled further back, opening the right atrial disk. After confirmation by echocardiography that the occluder was correctly positioned and there was no residual shunt through the ovoid fossa, a traction test was performed to secure the device, and the push rod turned down to release the occluder. After withdrawal of the push rod and sheath, the femoral vein was compressed to stop bleeding, the bandage being removed 6–8 h after surgery. For PFO patients with ASA, the operator will choose a larger-sized occluder to cover the ASA and repair it. All PFO closures were successful.

### Follow-up

After PFO closure, most patients were treated with dual antiplatelet therapy (aspirin 100 mg/day or aluminum-magnesium aspirin tablets MG/day plus clopidogrel hydrosulfate tablets 75 mg/day) for 6 months and antiplatelet therapy alone (aspirin 100 mg/day) for a further 3 months. The patients in the medication-only group received dual antiplatelet therapy for 3 months followed by antiplatelet therapy alone. The participants were followed up regularly (1, 3, 6, 12, and 24 months) at a cardiology or neurology clinic, no episodes of atrial arrhythmias, atrial tachycardia, or atrial flutter were documented on ECG. The end points were recurrent PFO-related ischemic stroke, including ischemic cerebral embolism and transient ischemic attack (TIA), and death from any cause during the 2 years of follow-up. All suspected recurrent stroke cases were referred to an independent, blinded endpoint-assessment panel consisting of two neurologists and one radiologist. After reviewing brain-imaging files, discharge summaries and neurological examination reports, the panel discussed each event according to the diagnostic criteria for PFO-related stroke and reached consensus before it was included in the primary outcome. The definition of recurrent cryptogenic stroke is: after the first cryptogenic stroke occurs, another stroke event occurs, and after detailed etiological examination (such as imaging examination, cardiac monitoring, etc.), the specific cause still cannot be clearly identified (28).

### Statistical analysis

All statistical analyses were performed using SPSS 26.0. Measurement data were tested for normality using the Shapiro–Wilk test, whereas normally distributed data are presented as mean ± standard deviation (X ± S). Differences between the PFO closure and medication-only groups were compared using two independent sample *t-*tests. Categorical variables are presented as absolute counts and percentages and the *χ*^2^ test was used to compare differences between the groups. Cox regression was performed to determine the factors associated with time to recurrent PFO-related ischemic stroke. The Cox regression model established by the Omnibus test was valid (*P* < 0.001), indicating that at least one independent variable in the model significantly affected the dependent variable (recurrent PFO-related ischemic stroke). *P* < 0.05 was considered to denote statistical significance.

## Results

The study comprised 207 patients. Their characteristics are described in Table 1. Of these patients, 120 (male 54.17%) underwent successful percutaneous PFO closure (PFO closure group). Most of this group also received oral antiplatelet therapy. The remaining 87 (male 56.32%) patients only received oral antiplatelet therapy. The prevalences of hypertension, diabetes mellitus, and smoking were 30.00%, 9.17% and 23.33%, respectively, in the PFO closure group, whereas these prevalences were 34.48%, 16.09%, and 33.33%, respectively, in the medication-only group. There were no significant differences between the two groups in these variables.

**Table 1:** Characteristics of study cohort.

Variable	PFO closure group (*n* = 120)	Medication-only Group (*n* = 87)	*P* Value
Demographic data			
Age (years)	46.94 ± 13.61	46.43 ± 12.97	0.784
Male	65 (54.17)	49 (56.32)	0.758
Hypertension	36 (30.00)	30 (34.48)	0.495
Diabetes mellitus	11 (9.17)	14 (16.09)	0.131
Current smoking	28 (23.33)	29 (33.33)	0.112
CHADS-VA score	0.42 ± 0.50	0.38 ± 0.49	0.631
Basic echocardiographic data			
LVIDd (mm)	29.08 ± 3.47	29.41 ± 3.24	0.476
LVIDs (mm)	47.33 ± 4.42	47.58 ± 4.15	0.561
FS (%)	38.10 ± 4.86	37.76 ± 4.61	0.539
EF (%)	66.46 ± 5.32	66.24 ± 5.11	0.603
LAAP (mm)	28.38 ± 2.77	28.14 ± 2.92	0.523
PFO anatomical features			
PFO width (mm)	1.81 ± 0.99	1.94 ± 0.93	0.361
PFO tunnel length (mm)	8.58 ± 3.43	9.52 ± 3.41	0.054
Right-to-left shunt grade			0.262
Small (1 to 9 microbubbles)	6 (5.00)	6 (6.90)	
Medium (10 to 30 microbubbles)	37 (30.83)	35 (40.23)	
Large (>30 microbubbles)	77 (64.17)	46 (52.87)	
Atrial septal aneurysm	24 (20.00)	16 (18.39)	0.772
Antiplatelet therapy			
At 3 months	95 (114/120)	93 (81/87)	0.573
At 6 months	93 (112/120)	90 (78/87)	0.355
At 9 months	89 (96/120)	87 (76/87)	0.149

Values are presented as mean ± SD or *n* (%).

LVIDd, left-ventricular end-systolic diameter; LVIDs, left-ventricular end-diastolic diamete; FS, fractional shortening; EF, ejection fraction; LAAP, left-atrial anteroposterior diameter; PFO, patent foramen ovale.

The anatomical features of the PFO in the closure and medication-only groups were as follows: maximum separation of PFO, 1.81 ± 0.99 mm vs. 1.94 ± 0.93 mm; length of PFO, 8.58 ± 3.43 mm vs. 9.52 ± 3.41 mm; and ASA with PFO, 20.00% vs. 18.39%. The prevalences of small, medium, and large right-to-left shunts were 5.00% vs. 6.90%, 30.83% vs. 40.23%, and 64.17% vs. 52.87%, respectively (Table 1). None of these anatomical features of the PFOs differed significantly between the two groups.

**Figure 4:**
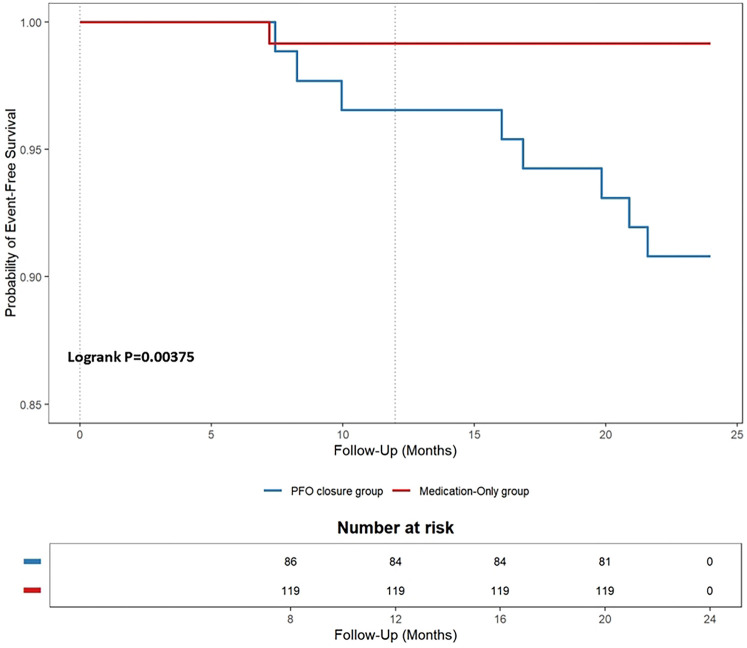
Kaplan–meier cumulative estimates of the primary endpoint in the patent foramen ovale (PFO) closure group versus the medication-only group.

We also compared follow-up data between the PFO closure and medication-only groups. During the 2-year follow-up period, the endpoint events detected were as follows (see Table 2 for details). One patient in the PFO closure group had a TIA (event rate, 0.83%). This patient had a long tunnel PFO (14.1 mm) (32). In the medication-only group, the following endpoint events occurred in eight patients (event rate, 9.20%): recurrent CS (three patients) and TIA (five patients). The PFO closure group had a lower rate of endpoint events than did the medication-only group, Kaplan–Meier curves showing that this difference was significant (log-rank *P* = 0.004, see Figure 4 for details).

**Table 2:** Characteristics of the study group during follow-up.

24-Mth outcome	PFO closure group (*n* = 120)	Medication-only group (*n* = 87)	*P* Value
Time of follow-up (mths)	23.86 ± 1.55	23.20 ± 3.04	0.064
All Endpoints	1 (0.83)	8 (9.20)	0.010
Ischemic cerebral embolism	0 (0.00)	3 (3.45)	0.402
Transient ischemic attack	1 (0.83)	5 (5.75)	0.028
Death	0 (0.00)	0 (0.00)	NA

Values are presented as mean ± SD or *n* (%).

PFO, patent foramen ovale.

As to the anatomical characteristics of PFO in the patients with recurrent PFO-related ischemic stroke, the proportion of PFOs with ASAs and large right-to-left shunts was higher in the medication-only group than in the patients without stroke. However, there were no significant differences in PFO width and tunnel length between these two groups of patients (see Table 3 for details). We therefore performed mixed effects Cox regression to determine the factors associated with recurrence of PFO-related stroke and TIA. According to this model (Table 4), ASA was independently associated with time to recurrent PFO-related ischemic stroke (B = 2.43, hazard ratio = 11.37; 95% CI: 1.53–85.14; *P* = 0.012）. That is, the risk of recurrent PFO-related stroke was 11.37 times higher in PFO patients with ASA than in those without ASA, whereas right-to-left shunt, PFO width, and tunnel length were not significantly associated with time to recurrent PFO-related ischemic stroke.

**Table 3:** Anatomical features of PFO in patients in the medication-only group who had recurrent ischemic strokes.

Variable	Recurrent ischemic stroke (*n* = 8)	Without recurrent ischemic stroke (*n* = 79)	*P* Value
Large Right-to-left shunt	8 (100.00)	38 (48.10)	0.040
Atrial septal aneurysm	7 (87.50)	9 (11.39)	0.000
PFO width (mm)	2.33 ± 0.92	1.90 ± 0.92	0.236
PFO tunnel length (mm)	10.66 ± 2.73	9.410 ± 3.46	0.257

Values are presented as mean ± SD or *n* (%).

PFO, patent foramen ovale.

**Table 4:** Associations between anatomical features of PFO and time to endpoints (multivariable analysis, mixed effects Cox eegression).

Variable	B	Hazard ratio (95% CI)	*P* Value
Right-to-left shunt	8.79	6,546.80 (0.00-Inf)	0.849
Atrial septal aneurysm	2.43	11.37 (1.53–85.14)	0.012
PFO width (mm)	0.49	1.63 (0.84–3.17)	0.146
PFO tunnel length (mm)	0.34	1.41 (0.95–1.00)	0.073

PFO, patent foramen ovale.

## Discussion

It is uncertain whether device closure or medical therapy is optimal for managing patients with PFO-associated stroke (16–18, 25). In this study, the rates of recurrent PFO-related ischemic stroke were significantly lower in patients who had undergone PFO closure plus antiplatelet therapy than in those who had received medical therapy alone (0.83% vs. 9.2%). We found associations between the anatomical features of PFO of ASA and large right-to-left shunts (“large PFO”) (23, 24). Further analysis showed that the presence of ASA significantly impacted the risk of stroke recurrence in patients with recent PFO-associated ischemic stroke. However, large PFOs were not independently associated with recurrent stroke.

The potential role of risk stratification based on the anatomical characteristics of PFO is unclear (25). However, previous studies have consistently demonstrated that the morphological features associated with PFO-associated stroke are large PFO, long tunnel, and ASA (23, 24, 33–35). In the present study our findings concerning the anatomic characteristics of PFO associated with recurrent PFO-related stroke were only partially consistent with these previously published findings.

Our procedure for measuring right-to-left shunting differed from those in previous studies. Previous studies have mostly used contrast TEE to assess right-to-left shunting (23, 24). However, many patients, especially stroke patients, cannot perform Valsalva maneuvers correctly during TEE because of aphasia, anosognosia, sedation, or the simple presence of an ultrasound probe in their throats (23, 24). The accuracy of assessment of right-to-left shunts with TTE is therefore poor (31). In the present study, we also used contrast TTE to assess right-to-left shunts and confirmed that large right-to-left shunts are associated with recurrent PFO-related strokes, consistent with previous results.

ASA, a localized, “saccular” deformity, is generally found in the central region of the undulating portion of the septum. The mechanism by which the presence of ASA affects the risk of PFO-related stroke in patients with PFO is not yet clear. The mechanism by which an atrial septal aneurysm (ASA) heightens the risk of recurrent PFO-related stroke remains incompletely understood. Besides channelling inferior-vena-cava blood toward the foramen ovale and keeping a large PFO open with every heartbeat (36, 37), ASA may promote recurrence through local endothelial trauma, blood stasis and *in situ* thrombogenesis. Repetitive flexion of the redundant septal membrane subjects the endothelium to shear stress and micro-tears, exposing collagen and tissue factor that foster platelet adhesion and thrombus formation within the PFO tunnel. Histological studies show thickened, neo-vascularised and inflamed tissue in ASA walls, creating a pro-thrombotic surface that can shed micro-emboli. Impaired regional atrial contractility and slowed left-atrial appendage flow further generate stasis, while associated Eustachian-valve remnants streamline venous flow toward the septum. These factors combine to sustain a hyper-coagulable milieu, explaining why patients with ASA plus large shunt exhibit the highest recurrence rates on medical therapy and derive the greatest absolute benefit from PFO closure (38). Because the two anatomical features of ASA and large PFO exist simultaneously, they do not have a synergistic effect in patients with recurrent stroke.

Long-tunnel PFOs are characterized by a significantly long (>8–10 mm) overlap between the septum primum and septum secundum. It has been shown that long-tunnel morphology increases the risk of clot formation (32). Blood stasis in the tunnel is a potential mechanism for PFO-induced thrombosis: one study found that long tunnels are an important risk factor in patients presenting with PFO-related stroke or TIA (34). However, we did not replicate these findings, presumably because our patients were receiving antiplatelet therapy.

Although only eight patients (approximately 9.2%) in the medical therapy only group had a recurrent PFO-related stroke, the proportion of recurrent PFO-related stroke in patients who are not taking antiplatelet drugs for a variety of reasons is likely to be higher. The morphological characteristics of PFO should be evaluated in these patients. Screening for the anatomical features of a large PFO or ASA may enable identification of the patients who would most benefit from PFO closure.

### Study strengths and limitations

Although the methodology and data of the study were not innovative, our findings addressed gaps in indications for closure of PFO. There has been very little research in China, despite Chinese patients accounting for more than 20% of patients globally. Therefore, our findings are valuable from the perspective of filling the gap in indications for PFO closure. This study is a retrospective study, the study subjects were the actual cases that occurred between January 2018 and December 2022, and the patient's subjective wishes and ethics need to be considered. There is a possibility of selection bias. Meanwhile, the limited number of endpoint events may affect the statistical power. In future research and clinical practice, we will continue to collect clinical data of patients with PFO-related stroke, to increase the sample size and enhance the statistical power of the results. In addition, the duration of follow-up was short. Currently, no data are available regarding the very long-term risk (>10 years) of stroke recurrence in patients with PFO-associated stroke receiving medical therapy. Thus, these patients should be followed up for longer to obtain the required data. We did not analyze 3D image features of PFO in this study. In a further study, we will add 3D imaging of PFOs to provide more information concerning indications for PFO closure.

## Conclusions

According to our findings, after 2 years of follow-up, the odds of recurrent PFO-related ischemic stroke are significantly lower in patients who have undergone PFO closure plus medical therapy than in those who have received medical therapy alone (0.83% vs. 9.2%). ASAs and large right-to-left shunts are associated with a high risk of recurrent stroke in PFO patients. These patients may derive the most benefit from PFO closure because such closure is associated with reduction in their high absolute risk.

## Data Availability

The raw data supporting the conclusions of this article will be made available by the authors, without undue reservation.
